# Allocation of Flood Drainage Rights in Watershed Using a Hybrid FBWM-Grey-TOPSIS Method: A Case Study of the Jiangsu Section of the Sunan Canal, China

**DOI:** 10.3390/ijerph19138180

**Published:** 2022-07-04

**Authors:** Xiaoyan Zhang, Juqin Shen, Fuhua Sun, Shou Wang, Shuxuan Zhang, Jian Chen

**Affiliations:** 1 College of Agricultural Science and Engineering, Hohai University, Nanjing 211100, China; jqshen@hhu.edu.cn (J.S.); fhsun@hhu.edu.cn (F.S.); wangshou201488@163.com (S.W.); zhangsxphd@gmail.com (S.Z.); 2 College of Business, Yancheng Teachers University, Yancheng 224007, China; 3 College of Civil Engineering, Nanjing Forestry University, Nanjing 210037, China; acme_chenjian@163.com

**Keywords:** flood drainage right, synergetic theory, fuzzy best–worst method, Grey-TOPSIS

## Abstract

In this study, an FDR allocation scheme based on synergetic theory was designed to alleviate the drainage conflicts caused by the grabbing of flood drainage rights (FDR) in each region of the basin. An FDR allocation index system was constructed by employing synergetic theory and following the principles of safety, equity, efficiency, and sustainability. A new multi-criteria decision-making method, called FBWM-Grey-TOPSIS, was developed, which is based on the integration of the fuzzy best–worst method (FBWM) and Grey-TOPSIS. Among them, the FBWM method was used to distinguish the importance of subsystems and order parameters, and the Grey-TOPSIS method is applied to obtain the optimal FDR assignment results. Taking the Jiangsu section of the Sunan Canal as an example, the FDRs of the four regions in the basin were allocated. The results reveal that the proportion of FDRs obtained in descending order is Changzhou (32.69%), Suzhou (24.88%), Wuxi (23.01%), and Zhenjiang (19.42%). In addition, the performance of the proposed method is demonstrated by sensitivity analysis and comparative analysis with the existing methods. The methodology and research results presented in this paper can help governments and agencies achieve a scientific allocation of FDR in watersheds, thus promoting harmonious watershed development.

## 1. Introduction

In recent years, as global temperatures continue to rise, the climate has become more abnormal and floods have occurred more frequently [1,2]. Floods not only result in great human suffering but also cause serious economic losses and ecosystem damage [3,4,5,6]. The sixth assessment report of the United Nations Panel on Climate Change (IPCC) states that global surface temperatures will continue to rise in the 21st century and that intense precipitation events are likely to become more frequent. The intensity of extreme daily precipitation events will increase by 7% for every 1 °C of global warming in the future (high confidence) [7]. Some scholars predict that global warming could amplify the risk of global flooding by 20 times by the end of the 21st century [8]. As can be seen, flood disasters have become one of the most severe social problems that humanity will have to face in the future [9].

In China, especially in the middle and lower reaches of the Yangtze River plain, due to abundant rainfall and a dense river network, flood disasters of different degrees frequently occur, which pose a significant threat to people’s lives, properties, and ecological environment [10,11]. Flooding has become a major constraint on China’s sustainable socio-economic development [12]. To reduce flood damage, many regions have invested a lot of human and material resources to build flood control facilities, and continuously improve their flood control standards to resist floods [13]. There is a growing recognition that flood protection facilities will always have a limited flood protection capacity and that their reinforcement and expansion cannot keep pace with the increasing intensity of floods, thus non-engineering measures are needed [14,15].

Once heavy rainfall occurs, it will inevitably lead to a significant increase in flood drainage demand in various regions of the basin. Flood disasters are inevitable when the flood drainage demand of the basin exceeds the flood control capacity. In China, the government finances flood control projects and owns the flood drainage rights (FDR) [16], which has led to these flood control works being treated as zero-price, free-use items for a long time [17]. FDR becomes a scarce resource during the flood season when the flood volume exceeds the flood control capacity of the basin [18]. Regions seek to maximize profits and plunder these scarce resources, which triggers drainage conflicts [19].

In past research efforts, many researchers have focused in various manners on the distribution of scarce resources such as FDR in the floodplain. The demand–supply mismatch triggered by scarce resources in floodplains can make managers’ decisions more difficult, and conflicts can be mitigated by designing allocation optimization models based on the identification of various policies [20,21]. For example, the redistribution scheme of wetland resources in floodplains under different scenarios is designed based on ecological crisis and flood risk perspectives [22], and a mixed Inexact-Quadratic fuzzy water resources management model for floodplains under sustainable development is developed [23]. The allocation of FDR is a way to mitigate flood hazards through policy management tools, so these studies provide a good reference for the allocation of FDR in floodplains. However, FDR is characterized by scarcity, public interest, social, and priority, and should be allocated and traded under the “quasi-market” mechanism [24]. In terms of allocation methods, a bi-level multi-objective programming model based on equity and efficiency perspectives can be developed to study the allocation of FDRs based on a “top-down” allocation model [25]. Some scholars have used the combined PSR model and entropy-based matching theory [26], fuzzy hierarchical analysis of environmental Gini coefficients [27], entropy weight TOPSIS model [10], and harmonious diagnostic model [28] to study the allocation of FDRs in watersheds, all of these methods have obtained better results of FDR allocation. Some researchers have also analyzed the pricing of FDR transactions [29,30]. The results of these investigations provide a good basis for this paper.

However, there are still some limitations in the current research on FDR allocation. The traditional FDR allocation indicator system is mostly built based on allocation principles, such as equity, efficiency, and sustainable development [27,28], with little attention to the evolutionary patterns of the subsystems and factors in the FDR allocation system, resulting in a lack of systematicity and completeness of the indicator system. In addition, the existing allocation methods, especially the frequently used AHF method, have high decision costs [31], do not consider the ambiguity of decision makers’ preferences in the decision-making process, and the accuracy and practicality of the allocation results need to be further improved [32,33]. In particular, the dynamic nature of flood hazard risk gives uncertainty and ambiguity to the continuous change of decision makers’ preferences. Meanwhile, the traditional TOPSIS, first proposed by Hwang and Yoon [34], is widely used, but it selects the optimal solution only based on the Euclidean distance, which may result in the inability to accurately judge the relative merits of the evaluation solution based on the relative closeness [35]. Therefore, we establish the FDR allocation index system based on the idea of synergistic theory. The best–worst method (BWM) constructed by Rezaei [36] is combined with the Grey-TOPSIS method as a way to overcome these issues. To better reflect the fuzziness and uncertainty of expert preferences, the traditional fuzzy preference relationship (FPR) is integrated into the BWM in the evaluation process to form the fuzzy BWM (FBWM) [32].

In summary, the purpose of this study is to integrate the FBWM method and Grey-TOPSIS method to develop a novel hybrid FBWM-Grey-TOPSIS method for watershed FDR distribution. Compared with other multi-criteria decision making (MCDM) methods, the FBWM-Grey-TOPSIS method has two distinct advantages. On the one hand, it describes the ambiguity and uncertainty of decision makers in the real world which makes the results close to reality [37]. On the other hand, the introduction of grey correlation improves the misclassification caused by a single distance and makes the results more reliable [38]. The relative merits of the solutions are identified more precisely, and the accuracy of the assignment results is improved. The proposed FBWM-Grey-TOPSIS method is applied to the FDR allocation problem of the Jiangsu section of the Sunan Canal in China during the flood season. During the flood season, especially when the flood drainage demand exceeds the regional flood drainage capacity, seeking a scientific and reasonable FDR allocation scheme under the permission and guidance of laws and regulations is an important means to control the flood drainage behavior of each region in the basin, and an important task to ensure the safety of each region. The methodology constructed in this study is expected to provide the best FDR allocation options for governments and watershed management agencies.

## 2. Materials and Methods

### 2.1. Research Area

**Figure 1:**
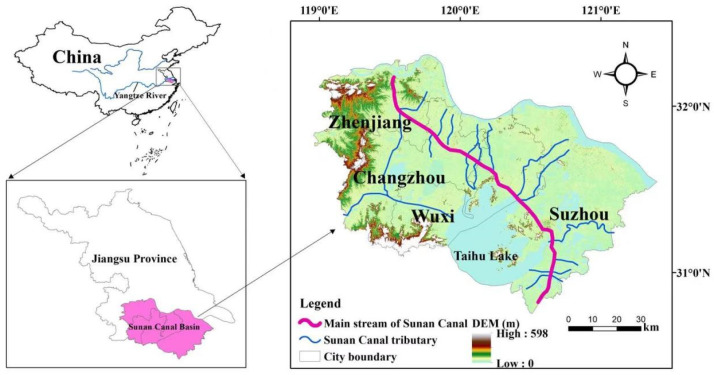
Administrative divisions of the Jiangsu section of the Sunan Canal.

The Sunan Canal, also known as the Jiangnan Canal, is one of the earliest formed sections of the Beijing-Hangzhou Grand Canal and is located in the Taihu Lake water network plain downstream of the Yangtze River. It mainly includes four cities in Jiangsu province and a small part of Shanghai city and Zhejiang province. The Jiangsu section of the Sunan Canal runs through the four economically developed cities of Zhenjiang, Changzhou, Wuxi, and Suzhou (Figure 1). It starts from the mouth gate of the Yangtze River Jianbi in Zhenjiang in the north and ends at the junction of Jiangsu and Zhejiang in the south at Yazi Dam, with a total length of about 212.5 km and an area of approximately 19,600 km^2^. It connects about 6000 km of waterways in the Taihu Lake area into a network, playing the role of water regulation and transshipment. In addition to being a “golden waterway” for shipping, it also has essential functions such as flood control, water drainage, irrigation and water supply, and landscape.

The Sunan Canal Basin is a humid northern subtropical climate zone, with distinct monsoonal characteristics and four distinct seasons. After the invasion of cold air in winter, there are many northerly winds and the weather is cold and dry. In the spring and summer transition, due to the warm and humid air currents moving north, cold and warm air currents encounter the formation of continuous cloudy rain, called “plum rain”, In summer, controlled by the subtropical high pressure, the weather is sunny and hot, and at the same time, it is often affected by tropical storms and typhoons, which easily form stormy and windy disaster weather. Rainfall in the basin is mainly concentrated from May to September, and flooding is frequent. In particular, the impact of the super El Niño event in 2016, the flooding along the Jiangsu section of the Sunan Canal is unusually severe, the Taihu Lake area a watershed mega-flood, the cumulative rainfall during the flood exceeded the same period in 1999, ranked first in history (1164 mm), the average surface rainfall during the rainy season is 2.4 times the average of the normal year. As a special “reservoir”, the canal is forced to accept the flood water from the cities along its route, which leads to Wuxi and Suzhou stations of the Sunan Canal reaching 5.28 m and 4.28 m respectively, with the water level breaking through the historical extreme again and continuing to exceed the warning level for a long time (27 to 58 days). Four cities suffered different degrees of flooding, and the flood disasters caused direct economic losses of 513 million yuan, 5262 million yuan, 409 million yuan, and 155 million yuan in Wuxi, Changzhou, Zhenjiang, and Suzhou, respectively.

During the flood season, the closer the city is to the upper reaches of the canal, the greater the impact of its drainage on floods. When a flood is released into a river from an upstream city, it does not affect the downstream city if the intensity of the flood does not exceed the flood control capacity of the river, but poses a threat to the downstream city if it exceeds the river’s flood control capacity. Therefore, drainage conflicts arise when the flood intensity exceeds the river’s flooding capacity. With the increasing drainage conflicts, the Water Resources Department of Jiangsu Province proposed to allocate the basin FDR to improve the canal’s drainage efficiency and reduce the flood disaster losses in the basin.

### 2.2. Methods

**Figure 2:**
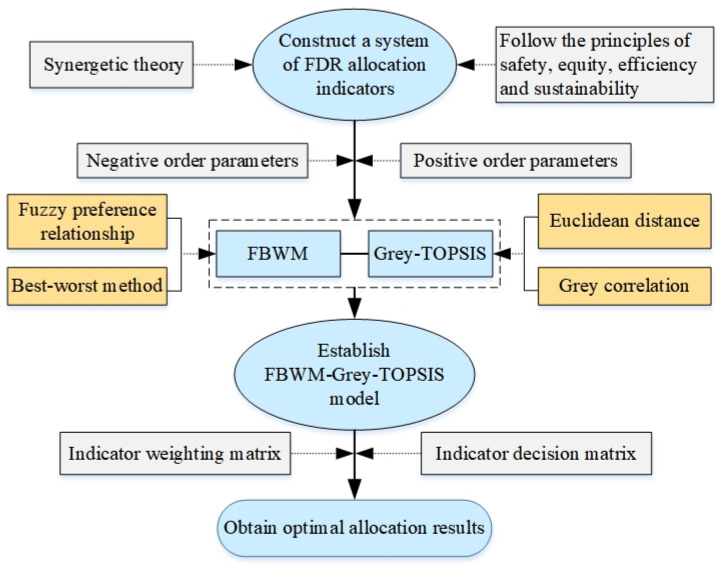
Flow chart of FDR allocation in a basin.

In this paper, the FBWM-Grey-TOPSIS method is proposed by combining FBWM with Grey-TOPSIS, and the method is applied to optimize FDR allocation of the Jiangsu section of the Sunan Canal in China. It is a novel MCDM approach that has never been used in the FDR field before and has unique advantages. The specific steps of the method are shown in Figure 2. Firstly, taking the synergetic theory as the basis and following the principles of safety, fairness, efficiency, and sustainable development, the FDR allocation indicator system including the economic subsystem, social subsystem, and natural environment subsystem is constructed. Secondly, the weight matrices of the order parameters are obtained by applying FBWM according to the fuzzy preferences of the experts. Finally, the normalized index data, i.e., the decision matrix and the weight matrix obtained by the FBMW method, are applied to the Grey-TOPSIS model to find the best results for the FDR allocation.

#### 2.2.1. Construction of Indicator System

Synergetic theory, which is mainly about the orderly evolution of systems and the coordinated development of subsystems, has been effectively applied in water resource allocation and evaluation [39,40,41]. The goal of FDR allocation is to coordinate the relationship between the drainage subjects, evolve the FDR system towards an orderly direction, and seek a reasonable FDR allocation scheme. Referring to the “social-economic-natural” composite system proposed by Wang et al. [42], the FDR system consists of three subsystems: economic, social, and natural environment, through competition and cooperation. Using hierarchy theory, a hierarchical structure is constructed by simplifying the complex system, which includes a target level (system), a criterion layer (subsystem), an indicator layer (order parameter), and an alternative layer (solution).

The order parameters are critical factors in the evolution of the FDR system and decisive indicators for FDR assignment. In the process of finding the order parameters, we not only combine the existing principles of scarce resource allocation and the characteristics of FDR allocation but also follow the principles of security, equity, efficiency, and sustainable development [43,44,45]. The specific meaning of each principle is as follows:

The principle of safety is the first principle to be followed in the allocation of FDR, which means that the number of FDRs in each area of the basin should be allocated scientifically and reasonably while avoiding casualties as much as possible. The principle of equity refers to the equitable distribution of FDR to all regions with full consideration of all influencing factors. The principle of efficiency refers to the full utilization of FDR, a scarce resource, and the ultimate goal of the allocation is to maximize the value of this resource. Based on following the above principles, by reviewing the literature, we identify that the FDR allocation indicator system contains 18 order parameters, as shown in Table 1.

(1) Economic subsystem

The economic subsystem plays a vital role in the FDR system to fully use FDR resources, improve drainage efficiency, and stabilize economic development. When the flood volume exceeds the drainage capacity of the basin, there is a certain competitive game between social stability and economic development [18]. Economic development potential reflects a region’s economic construction and future economic development capacity. Historical disaster losses represent a region’s interest in FDR allocation and can improve the rationality of FDR allocation. Therefore, the economic subsystem includes six order parameters: per capita GDP (s_11_), industrial added value (s_12_), per capita disposable income (s_13_), Engel coefficient (s_14_), flood direct economic loss (s_15_), and degree of industrial structure optimization (s_16_). We understand that indirect economic losses are perhaps greater than direct economic losses when floods occur; however, indirect economic losses are difficult to assess. There is no generally accepted quantitative method to assess it [48] and it is difficult to obtain reliable data, which is the reason why we only include direct economic losses from floods in our system. In addition, industrial structure optimization is the core variable explaining the economic development rate and development mode [46] and is an important ordinal covariate of economic subsystem development in FDR distribution, which is mainly measured by industrial structure rationalization and industrial structure upgrading. However, the value of this order parameter is difficult to account for directly, so we use it as a qualitative indicator whose value is determined through expert scoring. The first five order parameters are quantitative, and the last one is qualitative.

(2) Social subsystem

The social subsystem is an important driver for developing a synergistic FDR system that contributes to the social equity of FDR distribution. Population reflects how regions are affected by floods, with the more densely distributed regions being more affected by flood hazards. The strengthening of water conservancy facilities can expand the drainage capacity of the watershed, enhance drainage control, and thus improve drainage efficiency [10]. In addition, due to the differences in socioeconomic conditions and resource endowments among regions, social policy preferences should also be considered when allocating FDRs. The specific order parameters are population density (s_21_), employment rate (s_22_), urbanization rate (s_23_), drainage pipe length (s_24_), water construction investment (s_25_), and policy inclination (s_26_). In these order parameters, the first five are quantitative, and the last one is qualitative.

(3) Natural environment subsystem

The natural environment subsystem is the key factor affecting the balance of the whole drainage system. It is mainly influenced by the rainfall situation and the composition of the regional substratum on the storage status of precipitation, as well as the protection status of the ecological environment in the process of flood drainage. Due to the uneven spatial and temporal division of precipitation, the more abundant precipitation is, the greater the demand for FDR [28]. When rainfall exceeds the drainage capacity of a watershed, flood disasters occur and damage the ecosystem. The specific order parameters are annual rainfall (s_31_), built-up area (s_32_), green coverage (s_33_), sewage treatment rate (s_34_), water quality compliance rate in water functional areas (s_35_), and water resources development and utilization degree (s_36_). The first five of these order parameters are quantitative, and the last one is qualitative.

#### 2.2.2. Normalize the Raw Data

To eliminate the differences in measurement units and orders of magnitude between the raw data of each order parameter, we normalize the raw data. Let the original decision matrix $X_{ij}$ consists of n alternatives and m indicators.

(1) $$X_{ij}=\left[\begin{array}{l} \begin{array}{ccc} \begin{array}{cc} x_{11} & x_{21} \end{array} & \cdots & x_{1m} \end{array}\\ \begin{array}{ccc} \begin{array}{cc} x_{21} & x_{22} \end{array} & \cdots & x_{2m} \end{array}\\ \begin{array}{ccc} \begin{array}{cc} \vdots & \vdots \end{array} & \ddots & \vdots \end{array}\\ \begin{array}{ccc} \begin{array}{cc} x_{n1} & x_{n2} \end{array} & \cdots & x_{nm} \end{array} \end{array}\right]$$

If the order parameters are quantitative, they are standardized according to Equation (2) [49]. If the order parameters are qualitative and their values cannot be collected directly, their normalized values can be determined using the FBWM method [32]. Finally, the normalized decision matrix $X_{ij}^{\prime }$ is derived.

(2) $$X_{ij}^{\prime }=\left\{\begin{array}{l} \begin{array}{cc} \frac{x_{ij}}{\sum_{i=1}^{m}x_{ij}} & if\begin{array}{cc} x_{ij}\;is\;benefit-type & \end{array} \end{array}\\ \begin{array}{cc} \frac{\frac{1}{x_{ij}}}{\sum_{i=1}^{m}\frac{1}{x_{ij}}} & if\begin{array}{cc} x_{ij}\;is\;cost-type & \end{array} \end{array} \end{array}\right.$$

#### 2.2.3. Fuzzy Best–Worst Method (FBWM)

The BWM method is a vector-based comparison method proposed by Rezaei to overcome the inconsistency problem of comparison results in AHP [36]. It performs only reference comparisons with fewer comparisons and higher consistency in solving MCDM problems [50]. BWM has been demonstrated to be a reliable and effective method [31,51,52]. Subsequently, some scholars have extended BWM in theory and application to MCDM problems in fuzzy environments [32,33]. Xu et al. (2021) integrated the traditional fuzzy preference relationship (FPR) into BWM and proposed the FBWM method to address the fuzziness and intangibility in human judgment [32]. The method was applied to multilevel structured group decision-making problems. The relevant steps of the FBWM method are as follows [32].

Step I: Determine a set of indicators

Let $G=\left\{g_{1},g_{2},\cdot \cdot \cdot ,g_{n}\right\}$ be a set of indicators. Let an FPR $R={\left(r_{ij}\right)}_{n\times n}$ is a fuzzy set satisfying for any i,j⊂N, there is $r_{ij}\geq 0$. $r_{ij}$ indicates the degree of preference of an indicator $g_{i}$ over $g_{j}$. If $g_{i}$ is the best indicator or $g_{j}$ is the worst indicator, $r_{ij}$ is defined as a fuzzy reference comparison.

Step II: Select the best indicator ($g_{B}$) and the worst indicator ($g_{W}$)

The best indicator (e.g., most important and desirable) and the worst indicator (e.g., least important and desirable) of the decision environment are selected based on the experts’ opinions.

Step III: Execute fuzzy reference comparisons of the best indicator

A number between 0.5 and 0.9 is used to denote the fuzzy preference of the best indicator over all the other indicators. The fuzzy best vector (FBV) can be obtained: $R_{B}=(r_{B1},r_{B2},\cdots r_{Bn})$, where $r_{Bj}$ expresses the degree of fuzzy preference of the best indicator $g_{B}$ over $g_{j}$. Obviously, $r_{BB}=0.5$. When $r_{Bj}>0.5$, it means that the best indicator $g_{B}$ is better than $g_{j}$, and the bigger the value of $r_{Bj}$, the better $g_{B}$ is than $g_{j}$ [53].

Step IV: Execute the fuzzy reference comparisons of the worst indicator

A number between 0.5 and 0.9 is also used to denote the remaining fuzzy reference comparisons to obtain the fuzzy worst vector (FWV): $R_{W}={\left(r_{1W},r_{2W},\cdots r_{nW}\right)}^{T}$, where $r_{jW}$ indicates the degree of fuzzy preference $g_{j}$ over the worst indicator $g_{W}$. Obviously, $r_{WW}=0.5$. When $r_{jW}>0.5$ it means that $g_{j}$ is preferred $g_{W}$, and the bigger value of $r_{jW}$, the better $g_{j}$ is over $g_{W}$.

Step V: Derive the optimal fuzzy weights ($w_{1}^{*},w_{2}^{*},\cdots w_{n}^{*}$)

Let $w={\left(w_{1},w_{2},\cdots w_{n}\right)}^{T}(w_{i}>0,\sum_{i=1}^{n}w_{i}=1)$ be the weight vector and $w_{i}$ is the weight of $g_{j}$. If An FPR $R={\left(r_{ij}\right)}_{n\times n}$ has multiplicative consistency, then it means that $r_{ij}=\frac{w_{i}}{w_{i}+w_{j}},\forall i,j\in n$ is valid [54]. Thus, we have Equations (3) and (4).

(3) $$r_{Bj}=\frac{w_{B}}{w_{B}+w_{j}}$$

(4) $$r_{jW}=\frac{w_{j}}{w_{j}+w_{W}}$$

To make Equations (3) and (4) always valid for all *j*, a solution, which satisfies the minimization of the maximum absolute differences $\left|\frac{w_{B}}{w_{B}+w_{j}}-r_{Bj}\right|$ and $\left|\frac{w_{j}}{w_{j}+w_{W}}-r_{jW}\right|$ for all *j*, should be found [32]. As a result, the linear programming Equation (5) for determining the optimal fuzzy weights can be obtained as follows:

(5) $$\begin{array}{c} \begin{array}{cc} \;\;\;\;\;\;\;\;\;\min & \max\left\{\left|\frac{w_{B}}{w_{B}+w_{j}}-r_{Bj}\right|,\left|\frac{w_{j}}{w_{j}+w_{W}}-r_{jW}\right|\right\} \end{array}\\ s.t.\left\{\begin{array}{l} \sum_{j=1}^{n}w_{j}=1\\ w_{j}\geq 0,for\begin{array}{cc} all\begin{array}{cc} j & \end{array} & \end{array} \end{array}\right. \end{array}$$

Equation (5) can be transferred into the following nonlinear constrained optimization Equation (6).

(6) $$\begin{array}{l} \min\begin{array}{cc} \xi & \end{array}\\ s.t.\left\{\begin{array}{l} \left|\frac{w_{B}}{w_{B}+w_{j}}-r_{Bj}\right|\leq \xi ,for\begin{array}{cc} all\begin{array}{cc} j & \end{array} & \end{array}\\ \left|\frac{w_{j}}{w_{j}+w_{W}}-r_{jW}\right|\leq \xi ,for\begin{array}{cc} all\begin{array}{cc} j & \end{array} & \end{array}\\ \sum_{j=1}^{n}w_{j}=1\\ w_{j}\geq 0,for\begin{array}{cc} all\begin{array}{cc} j & \end{array} & \end{array} \end{array}\right. \end{array}$$

For Equation (6), we can apply MATLAB for programming to obtain the best fuzzy weights ($w_{1}^{*},w_{2}^{*},\cdots w_{n}^{*}$) and $\xi^{*}$.

Finally, the consistency ratio (CR) needs to be calculated. The CR is an important indicator to test the degree of consistency between two comparisons [33]. As described above, 0.9 is the maximum possible value of $r_{BW}$. There will be inconsistency with the fuzzy pairwise comparison when $\frac{r_{Bj}}{r_{jB}}\frac{r_{jW}}{r_{Wj}}$ is higher or lower than $\frac{r_{BW}}{r_{WB}}$. This inequality will reach a maximum when $\frac{r_{Bj}}{r_{jB}}=\frac{r_{jW}}{r_{Wj}}=\frac{r_{BW}}{r_{WB}}$ [55], and at this point we obtain ξ.

It is also known that $\frac{\frac{w_{B}}{w_{B}+w_{j}}}{1-\frac{w_{B}}{w_{B}+w_{j}}}\frac{\frac{w_{j}}{w_{j}+w_{W}}}{1-\frac{w_{j}}{w_{j}+w_{W}}}=\frac{w_{B}}{w_{j}}\frac{w_{j}}{w_{W}}=\frac{w_{B}}{w_{W}}$, Therefore, when the maximum inequality appears, Equation (7) can be obtained as follows.

(7) $$\left(\frac{r_{Bj}}{r_{jB}}-\xi \right)\times \left(\frac{r_{jW}}{r_{Wj}}-\xi \right)=\left(\frac{r_{BW}}{r_{WB}}+\xi \right)$$

The minimum fuzzy consistency satisfies $\frac{r_{Bj}}{r_{jB}}=\frac{r_{jW}}{r_{Wj}}=\frac{r_{BW}}{r_{WB}}$, from which the following equation can be obtained:

(8) $$\left(\frac{r_{BW}}{r_{WB}}-\xi \right)\times \left(\frac{r_{BW}}{r_{WB}}-\xi \right)=\left(\frac{r_{BW}}{r_{WB}}+\xi \right)$$

Equation (8) can be expanded as follows:

(9) $$\xi^{2}-\left(1+2\frac{r_{BW}}{r_{WB}}\right)\xi +\left({\left(\frac{r_{BW}}{r_{WB}}\right)}^{2}-\frac{r_{BW}}{r_{WB}}\right)=0$$

By solving Equation (9), the maximum possible value of ξ can be found. These maximum values are used as the consistency index (CI) of FBWM, as shown in Table 2. The CR is defined as follows:

(10) $$CR=\frac{\xi^{*}}{CI}$$

where $\xi^{*}$ is obtained by solving Equation (6) and CI is the maximum ξ, as Table 2 shown (for more details see Xu et al., 2021). Equation (10) shows that the closer $\xi^{*}$ is to 0, the smaller the value of the CR is and the higher the reliability of pairwise comparisons.

#### 2.2.4. Grey-TOPSIS

The TOPSIS method, also known as the two-base method, is an MCDM method developed by Hwang and Yoon (1981) and has been widely used in academia [56]. This method analyzes the relative merits of the evaluated objects by ranking all alternatives by using their distances from positive ideal solutions (PIS) and negative ideal solutions (NIS) as evaluation criteria [57,58]. However, when using traditional TOPSIS, the distance of the option from PIS and NIS is estimated based on the Euclidean distance. In this case, there may be a situation where the evaluation scheme is close to the PIS and NIS, resulting in the inability to accurately judge the relative merits of the evaluation scheme based on the relative closeness, which affects the reliability of the evaluation results [35,59]. Therefore, we proposed the Grey-TOPSIS technique using two distances, i.e., Euclidean distance and grey correlation. It can effectively identify the relative position relationship between evaluation objects and improve the accuracy of evaluation. The procedure is as follows:

(1) Build the normalized weighted decision matrix

Combining the normalized decision matrix $X_{ij}^{\prime }$ obtained by Equation (2) and the optimal fuzzy weight matrices $w_{j}^{*}$, we can obtain the normalized weighted decision matrix Z.

(11) $$Z={\left(z_{ij}\right)}_{n\times m}={\left(X_{ij}^{\prime }w_{j}^{*}\right)}_{n\times m}$$

where $X_{ij}^{\prime }$ is the normalized data value, $w_{j}^{*}$ is the weight value of the indicator $g_{j}$, and $z_{ij}$ is the normalized weighted indicator value.

(2) Determine the positive and negative ideal solutions $z_{i}^{+}$ and $z_{i}^{-}$

(12) $$z_{i}^{+}=\left\{\begin{array}{l} \left\{\max z_{ij}\left|i=1,2,\cdots ,m=\left\{z_{1}^{+},z_{2}^{+},\cdots z_{m}^{+}\right\}\right.\right\}z_{i}\;is\;positive\;indicator\\ \left\{\min z_{ij}\left|i=1,2,\cdots ,m=\left\{z_{1}^{-},z_{2}^{-},\cdots z_{m}^{-}\right\}\right.\right\}z_{i}\;is\;negative\;indicator \end{array}\right.$$

(13) $$z_{i}^{-}=\left\{\begin{array}{l} \left\{\min z_{ij}\left|i=1,2,\cdots ,m=\left\{z_{1}^{-},z_{2}^{-},\cdots z_{m}^{-}\right\}\right.\right\}z_{i}\;is\;positive\;indicator\\ \left\{\max z_{ij}\left|i=1,2,\cdots ,m=\left\{z_{1}^{+},z_{2}^{+},\cdots z_{m}^{+}\right\}\right.\right\}z_{i}\;is\;negative\;indicator \end{array}\right.$$

(3) Calculate Euclidean distance and grey correlation

We can use Equation (14) to calculate the Euclidean distance $d_{i}^{+}$ and $d_{i}^{-}$, respectively.

(14) $$\begin{array}{l} d_{i}^{+}=\sqrt{{\sum_{j=1}^{m}\left(z_{ij}-z_{j}^{+}\right)}^{2}}\\ d_{i}^{-}=\sqrt{{\sum_{j=1}^{m}\left(z_{ij}-z_{j}^{-}\right)}^{2}} \end{array}$$

The grey correlation degree is calculated according to Equations (15) and (16).

(15) $$\begin{array}{l} v_{ij}^{+}=\frac{\underset{i}{\min}\;\underset{j}{\min}\left|z_{ij}-z_{j}^{+}\right|+\rho \;\underset{i}{\max}\;\underset{j}{\max}\left|z_{ij}-z_{j}^{+}\right|}{\left|z_{ij}-z_{j}^{+}\right|+\rho \;\underset{i}{\max}\;\underset{j}{\max}\left|z_{ij}-z_{j}^{+}\right|}\\ v_{ij}^{-}=\frac{\underset{i}{\min}\;\underset{j}{\min}\left|z_{ij}-z_{j}^{-}\right|+\rho \;\underset{i}{\max}\;\underset{j}{\max}\left|z_{ij}-z_{j}^{-}\right|}{\left|z_{ij}-z_{j}^{-}\right|+\rho \;\underset{i}{\max}\;\underset{j}{\max}\left|z_{ij}-z_{j}^{-}\right|} \end{array}$$

where $v_{ij}^{+}$ and $v_{ij}^{-}$ are the grey correlation coefficients, ρ is the resolution factor, ρ∈[0,1], and usually ρ=0.5.

(16) $$\begin{array}{l} v_{i}^{+}=\frac{1}{m}\sum_{j=1}^{m}v_{ij}^{+}\\ v_{i}^{-}=\frac{1}{m}\sum_{j=1}^{m}v_{ij}^{-} \end{array}$$

where $v_{i}^{+}$ and $v_{i}^{-}$ are the grey correlations between PIS and NIS, respectively.

(4) Construct Grey-Euclidean Distance Measures

After dimensionless processing of the obtained $d_{i}^{+}$,$d_{i}^{-}$, $v_{i}^{+}$, and $v_{i}^{-}$, we obtain $D_{i}^{+}$, $D_{i}^{-}$, $V_{i}^{+}$, and $V_{i}^{-}$, respectively. The calculation formula is as follows.

(17) $$\left.\begin{array}{l} D_{i}^{+}=D_{i}^{+}/\max D_{i}^{+}\\ D_{i}^{-}=D_{i}^{-}/\max D_{i}^{-}\\ V_{i}^{+}=V_{i}^{+}/\max V_{i}^{+}\\ V_{i}^{-}=V_{i}^{-}/\max V_{i}^{-} \end{array}\right\}$$

After merging the determined dimensionless weighted Euclidean distance and the grey correlation, two new distance measures $Y_{i}^{+}$ and $Y_{i}^{-}$ are defined, as shown in Equation (18)

(18) $$\begin{array}{l} Y_{i}^{+}=\alpha D_{i}^{-}+\beta V_{i}^{+}\\ Y_{i}^{-}=\alpha D_{i}^{+}+\beta V_{i}^{-} \end{array}$$

where α and β are the decision maker’s preferences, α+β=1. We have no subjective preference for the two methods, so α=β=0.5.

(5) Calculate the relative closeness

Using Equation (18), the relative closeness is calculated as follows:

(19) $$A_{i}=\frac{Y_{i}^{+}}{Y_{i}^{-}+Y_{i}^{+}}$$

(6) Calculate FDR allocation ratio

(20) $$\beta_{i}=\frac{A_{i}}{\sum_{i=1}^{n}A_{i}}$$

where $\beta_{i}$ is the proportion of FDR allocated in region *j*, $\sum_{j=1}^{m}\beta_{i}=1$.

## 3. Results

### 3.1. Indicator Normalization Results

Since a watershed mega-flood occurred in the Sunan Canal in 2016 and the water level exceeded the historical extreme, we analyzed it as a typical year. There were 15 quantitative order parameters in the FDR allocation indicator system in this paper. Their original values were obtained from the Jiangsu Statistical Yearbook, Jiangsu Water Resources Yearbook, and Jiangsu Water Resources Bulletin in 2017. These data were considered credible as publicly available in both Chinese and English public versions. Considering that the order parameter statistics had inconsistent characteristic scales (units) in different dimensions, the quantitative order parameters of the four cities were normalized by Equation (2).

**Figure 3:**
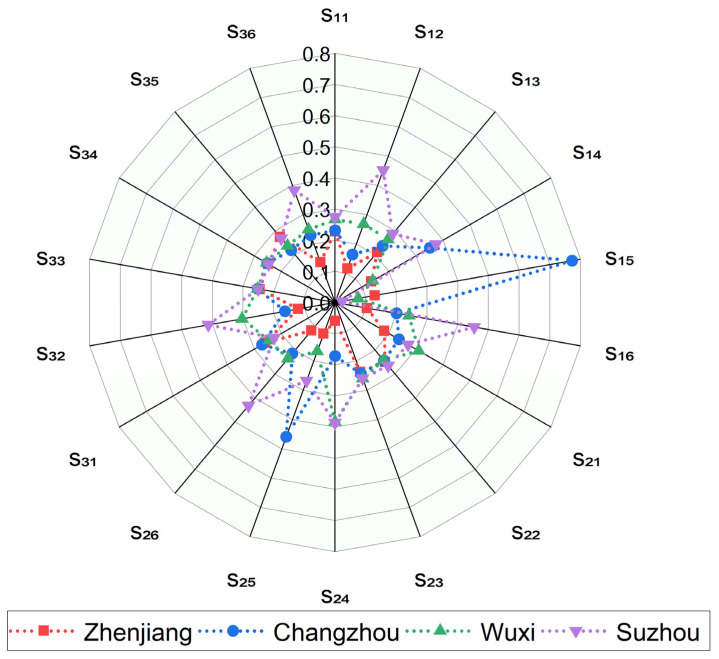
Normalized values of each order parameter for the four cities.

Meanwhile, there were three qualitative order parameters, namely degree of industrial structure optimization (s_16_), policy inclination (s_26_), and water resources development and utilization degree (s_36_). Since the values of these three qualitative order parameters were not obtained directly, we used the FBWM method to determine their normalized values [32]. Figure 3 shows the normalized values of each order parameter for these four cities.

According to Figure 3, Suzhou and Changzhou performed better on Engel coefficient (s_14_), while Wuxi and Zhenjiang were worse. Changzhou had the highest flood direct economic loss (s_15_), while the other three cities had lower values, especially Suzhou, whose value was much lower than Changzhou. This indicates that Changzhou is at greater risk of flooding than other cities because it will experience heavy rainfall that occurs once every 100 years, resulting in water levels well above warning levels. Wuxi had the largest value on population density (s_21_), while Zhenjiang had the smallest. The distribution of per capita GDP (s_11_), per capita disposable income (s_13_), employment rate (s_22_), urbanization rate (s_23_), annual rainfall (s_31_), green coverage (s_33_), sewage treatment rate (s_34_), and water quality compliance rate in water functional areas (s_35_) were relatively average. Changzhou had the best performance on water construction investment (s_25_), whereas Zhenjiang had the least. The remaining order parameters were similarly distributed, with Suzhou being the largest and Zhenjiang the smallest.

### 3.2. Determination of Indicator Weights

To obtain the weights of each subsystem and order parameter, we interviewed five experts in the field of FDR, respectively. These experts had long-term experience in FDR research or work in related government departments, mainly from Jiangsu Provincial Water Resources Department, Jiangsu Provincial Flood and Drought Control Command, and Hohai University.

The weight vectors of each subsystem and order parameter were determined separately using the FBWM method. According to the idea of the FBWM method, five experts first selected the best subsystem (or order parameter) and the worst subsystem (or order parameter). Then, the experts determined the fuzzy preferences of the best subsystem (or order parameter) over all the other subsystem (or order parameter) and the other subsystem (or order parameter) over the worst subsystem (or order parameter) using a number from 0.5 to 0.9, with 0.5 indicating equal importance and 0.9 indicating extreme importance. The fuzzy best vector $FBV^{\left(m\right)}$ and fuzzy worst vector $FWV^{\left(m\right)}$ provided by $e_{m}(m=1,2,3,4,5)$ are shown in Table 3.

For the subsystem (s_1_–s_3_), $FBV^{\left(1\right)}$ (0.5, 0.7, 0.6) and $FWV^{\left(1\right)}$ (0.7, 0.5, 0.6)^T^ of the first expert (*e*_1_) were used as an example to analyze the solving process. By substituting the vector elements into Equation (6), then Equation (21) was deduced.

(21) $$\begin{array}{l} \min\xi^{\left(1\right)}\\ s.t.\left\{\begin{array}{l} \left|\frac{w_{1}^{\left(1\right)}}{w_{1}^{\left(1\right)}+w_{2}^{\left(1\right)}}-0.7\right|\leq \xi^{\left(1\right)}\\ \left|\frac{w_{1}^{\left(1\right)}}{w_{1}^{\left(1\right)}+w_{3}^{\left(1\right)}}-0.6\right|\leq \xi^{\left(1\right)}\\ \left|\frac{w_{3}^{\left(1\right)}}{w_{3}^{\left(1\right)}+w_{2}^{\left(1\right)}}-0.6\right|\leq \xi^{\left(1\right)}\\ \begin{array}{c} w_{1}^{\left(1\right)}+w_{2}^{\left(1\right)}+w_{3}^{\left(1\right)}=1\\ w_{1}^{\left(1\right)}\geq 0,w_{2}^{\left(1\right)}\geq 0,w_{3}^{\left(1\right)}\geq 0 \end{array} \end{array}\right. \end{array}$$

We used the program written in MATLAB to solve the Equation (21) and thus obtained the weight vector of each subsystem and $\xi^{*1}$. The results were: $w_{}^{*(1)}$ = (0.478, 0.207, 0.315), $\xi^{*(1)}=0.003$, and CR = 0.0028/1.63 = 0.004. The value of CR was very close to zero, indicating a high degree of consistency in the comparison.

Similarly, the rest of the other results were obtained, as shown in Table 4 and Table 5, respectively. For simplicity, we assigned the same importance to all experts and used a simple averaging operator to obtain the weights of the subsystems and order parameters.

According to Table 4, the social subsystem plays the most significant role in the FDR allocation process with a weight of 0.434, followed by the economic subsystem with a weight of 0.374, and the natural environment subsystem is the least important with a weight of 0.192. This is because ensuring social security and stability in the region is the primary factor to be considered when allocating FDRs [60]. The stable development of regional social security is the basis for prosperous economic development. People pursue ecological sustainability, under social stability and rapid economic development. The value of CRs is very close to zero and has a high consistency.

Table 5 shows the local weights and consistency ratios of the corresponding order parameters in each subsystem. The values of CRs are all very close to zero, indicating a high degree of consistency. In the economic subsystem, the order parameter flood direct economic loss (s_15_) has a maximum weight of 0.261. It is because historical flood losses reflect the direct losses of an area affected by flood hazards in the past, so, the larger the value of s_15_, the more FDR is needed. The second is the per capita GDP (s_11_), because the higher the per capita GDP, the higher the cost of flood disaster recovery. Lower weights are given to the Engel coefficient (s_14_) and the degree of industrial structure optimization (s_16_).

In the social subsystem, population density (s_21_) has the highest weight value of 0.259. Because the safety of human life is the first principle of FDR allocation, the higher the population density, the greater the exposure to flood hazards. It is followed by the urbanization rate (s_23_), with a weight of 0.174. In contrast, drainage pipe length (s_24_) and employment rate (s_22_) were assigned lower weights.

In the natural environment subsystem, the annual rainfall (s_31_) is ranked first in terms of weight, and the higher the rainfall, the higher the possibility of forming floods and the greater the flood hazard. It is followed by greenery coverage (s_33_) and water resources development and utilization degree (s_36_), and the least weighted is the sewage treatment rate (s_34_).

After obtaining the local weights of the order parameters in each subsystem, the global weights of order parameters are obtained by multiplying the local weights by the weights of subsystems, as shown in Table 6.

As can be seen from Table 6, the social subsystem has the largest weight, giving relatively large weights to its global order parameters, followed by the economic subsystem and the natural environment subsystem. Among all order parameters, population density (s_21_) is ranked first with a global weight of 0.112, indicating that the primary factor in FDR allocation is securing life. The weights of flood direct economic loss (s_15_) and per capita GDP (s_11_) are ranked second and third, respectively. If a region has higher flood direct loss, the region suffers more flood damage during the flood season, and the demand for FDR is higher. Similarly, when a region has a high level of economic development, the same amount of flooding causes more damage to the area and is more expensive to repair. The weights of Water quality compliance rate in water functional areas (s_35_) and sewage treatment rate (s_34_) are ranked last and have a relatively weak impact on the FDR allocation.

### 3.3. Allocation Result of FDR

**Figure 4:**
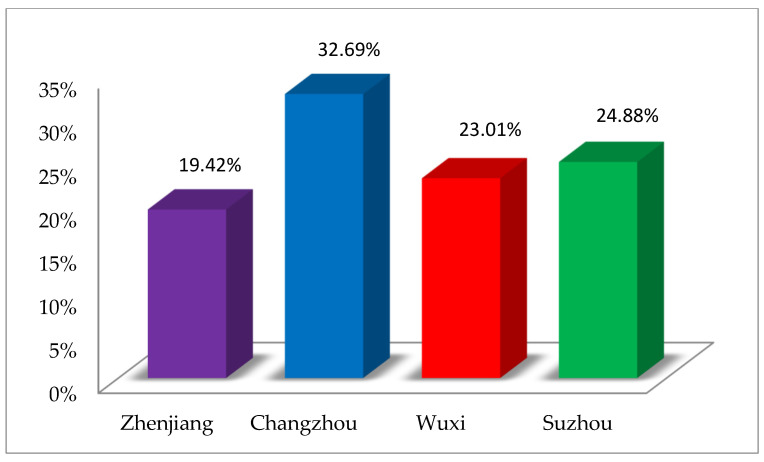
Result of FDR allocation scheme.

After obtaining the weights of the order parameters and normalizing the raw data, we measured the scores of the FDR allocation alternatives for the Jiangsu section of the Sunan Canal in China using the Grey-TOPSIS method. The result of the optimal FDR assignment is shown in Figure 4.

According to Figure 4, the results of FDR allocation in descending order are Changzhou (h_2_), Suzhou (h_4_), Wuxi (h_3_), and Zhenjiang (h_1_), with weights of 32.69%, 24.88%, 23.01%, and 19.42%, respectively. Changzhou City is located in the midstream section of the canal, and although it is not the most economically developed area, it has the largest FDR allocation ratio of the four cities, which means it can drain more flood water into the river to reduce its flood pressure. This is related to the fact that it faces the greatest risk of flooding disasters. Changzhou’s value of the most important factor, population density (s_21_), is less different from the other cities, while the value of the second-ranked factor, flood direct economic loss (s_15_), is the largest and much larger than the other three cities (as shown in Figure 4). Similarly, Changzhou performs significantly better than other cities in terms of water construction investment (s_25_), which is an important influencing factor for FDR allocation. It is also the reason why Suzhou and Wuxi cities are larger than Changzhou in other indicators but allocate a smaller percentage of FDR than Changzhou.

Wuxi City is downstream of Changzhou, and the flood water discharged from Changzhou will inevitably affect it. At the same time, the flood damage caused by the stronger rainfall in Wuxi is also greater, and more drainage rights need to be allocated. Suzhou is situated downstream of the canal, and although it receives the least amount of rainfall, it is most threatened by upstream drainage. Meanwhile, it has the most developed economy, and many indicators are better than those of other cities, so the proportion of drainage rights allocated is only less than that of Changzhou. Zhenjiang City is located in the upstream section of the canal with relatively poor performance on most indicators and therefore has the smallest share in the FDR allocation. This is also consistent with the fact that Zhenjiang has only one major outlet, the Jianbi pumping station, which means that there is less room for drainage regulation through manual pumping stations.

It can be seen that the coordinated development of the social, economic, and natural environment is the foundation for FDR allocation. In particular, factors such as population density, historical flood damage, and rainfall play an important guiding role in FDR allocation. To reduce flood damage losses for the entire basin, the proportion of the upstream Zhenjiang would be reduced in the FDR allocation plan if discharging flood water from the upstream Zhenjiang into the canal causes more damage to the downstream Changzhou area than if the flood water stays in the Zhenjiang area. The determination of the optimal allocation ratio of FDR is conducive to regulating the order of drainage during floods. It is also conducive to clarifying the main responsibility of compensation after activating the flood storage and stagnation zone in flood control dispatching, and promoting the formation of a situation of “who benefits and who compensates”. The government should actively explore the FDR allocation mechanism, weighing the needs, advantages, and benefits of each region.

## 4. Discussions

### 4.1. Sensitivity Analysis

**Figure 5:**
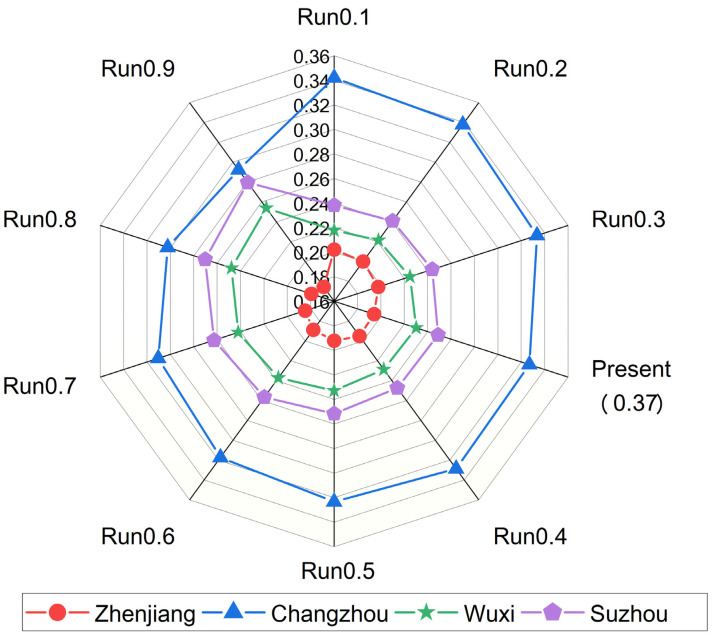
Sensitivity analysis results.

Sensitivity analysis helps reduce uncertainty in parameters and perceptions and plays an important role in determining the robustness of the solution [61]. The primary purpose of sensitivity analysis is to find the stability of the optimal solution under parameter variations [62]. Figure 5 shows that Changzhou has the largest proportion in the FDR distribution results, thereby influencing the proportion of other cities. Since small changes in indicator weights may make significant changes in the final ranking [63], sensitivity analysis can test the bias and robustness of the results by changing the information about the decision maker’s fuzzy preferences [64]. When the weights of the social subsystem vary between 0.1 and 0.9, the weights of the other subsystems vary accordingly, as shown in Table 7. At the same time, the optimal FDR assignment scheme is also changed, and the corresponding allocation results are shown in Figure 5. Due to space constraints, details about the variation of order parameter weights and rankings are not shown in this study, interested readers can contact us.

Figure 5 shows that the ranking of FDR proportions is relatively stable across the four regions (i.e., the alternatives). Although there are some variations among regions, several experiments have shown that the FDR allocation ratio fluctuates only slightly up and down among regions. When the weight of the social subsystem gradually increases from 0.1 to 0.9, Changzhou (h_2_) is consistently ranked first, and Zhenjiang (h_1_) is ranked last in terms of the FDR allocation ratio. At this time, the ranking of the alternatives remains unchanged always as Changzhou (h_2_) > Suzhou (h_4_) > Wuxi (h_3_) > Zhenjiang (h_1_). This result suggests that the social subsystem is the fundamental element of the current FDR allocation in the basin. The optimal allocation scheme derived by the FBWM-Grey-TOPSIS method is robust, and the final choice is not affected despite changes in the fuzzy preference information of the decision maker for the subsystem.

### 4.2. Comparisons with Other Methods

The FDR allocation method is critical because it can change the FDR allocation result. The results of FBWM-Grey-TOPSIS, FBWM-TOPSIS, and three single indicator assignment models are compared and analyzed, and the results are presented in Table 8.

According to Table 8, the “PAG” model only considers the influence of a single factor, so the distribution results are highly biased and difficult to be accepted by the regions. For example, the population density-based allocation result, considering only the population density factor, Wuxi’s population density exceeds Suzhou’s, resulting in its allocation of a higher percentage of FDRs than Suzhou. In reality, Suzhou is higher than Wuxi in terms of economic level and regional area, so the allocation result based on population density will inevitably lead to strong opposition from Suzhou.

The ranking of the four cities in the FBWM-TOPSIS-based allocation scenario is consistent with the FBWM-Grey-TOPSIS results, yet it is clear that there is a wide gap in the percentage of cities allocated. The allocation proportion of Changzhou City is much higher than that of Zhenjiang City, with a higher proportion of 35.93%. The result is bound to be resisted by Zhenjiang, so this program lacks fairness and has very low practicality. The differences in FDR allocation percentages for the four cities in the FBWM-Grey-TOPSIS-based allocation scheme are relatively small, with Zhenjiang’s percentage being increased to 19.42%, which indicates that it is allowed to discharge more flood water into the canal. The result is consistent with the reality that the Sunan Canal flows through a part of Zhenjiang with less demand for FDR. Therefore, the allocation result is more reasonable and easily accepted, which can alleviate the conflict areas between drainage.

By comparing the results of different allocation methods, it is found that the FDR allocation method pursuing a single objective is simple and easy to implement and can achieve certain drainage objectives. However, it has some limitations and is likely to cause dissatisfaction among parties with impaired interests, which is not conducive to the harmonious development of the watershed. In practical applications, FDR allocation is a complex process pursuing multiple objectives and dimensions, requiring comprehensive consideration of various factors.

According to the comparison results, our proposed FBWM-Grey-TOPSIS approach has the highest scientific validity. The approach makes the allocation results more reliable and acceptable to the regions by introducing the FBWM method and improving the traditional TOPSIS technique. Meanwhile, the proposed method is simple to operate, has low computational complexity, and yields more consistent results, which can better reduce decision costs. The methods and results of this study provide a good reference for FDR allocation, but further refinement is needed in the actual application process, and comprehensive analysis of rainfall, water, Engineering status, and socio-economic development of each region is required. We hope that governments and institutions will pay attention to the approach of this paper and actively explore the allocation system of FDR.

## 5. Conclusions

To alleviate drainage conflicts and reduce overall flood hazard losses in the watershed, this paper investigates the watershed FDR allocation problem. Firstly, based on the synergetic theory, the FDR allocation indicator system is constructed with the economic subsystem, social subsystem, and natural environment subsystem as the core. Secondly, the FBWM-Grey-TOPSIS method is proposed by integrating FBWM, a frontier method in MCDM, with Grey-TOPSIS. FBWM mothed can resolve the ambiguity and uncertainty in the real environment by incorporating FPR into BWM and obtaining the weights of the indicators. Grey-TOPSIS method, which incorporates Euclidean distance and grey correlation, is used to obtain a more reliable optimal allocation result. Finally, a case study of the Jiangsu section of the Sunan Canal in China is conducted by applying the FDR allocation indicator system and the FBWM-Grey-TOPSIS method. The distribution results show that the percentage of FDR received by Changzhou, Suzhou, Wuxi, and Zhenjiang are 32.69%, 24.88%, 23.01%, and 19.42%, respectively. The result is consistent with the objective of FDR allocation, which fully takes into account the differences in a regional social, economic, and natural environment. It provides guidance and reference for the government to use non-engineering means to improve the flood control system.

The novelty of this research is the combination of these individual methods to obtain the optimal FDR assignment scheme. The FBWM-Grey-TOPSIS method, as a novel MCDM method, can effectively deal with the uncertainty in resource allocation and applies not only to the watershed FDR allocation problem but also to other fields, such as water resources allocation. However, there is room for further improvement in our approach. For example, in the actual FDR allocation process, decision-makers may face more complex uncertainties. To better describe this realistic behavior, more complex fuzzy linguistic environments such as hesitant fuzzy linguistic environments, and type-2 fuzzy environments can be introduced in future studies [58].

## Figures and Tables

**Table 1:** FDR allocation indicator system.

Target Layer (System)	Criteria Layer (Subsystem)	Indicator Layer (Order Parameter)	Indicator Meaning	Nature	References
FDR allocation system	Economic subsystem (s_1_)	Per capita GDP (s_11_)	The relationship between regional GDP and resident population	+	[25,27]
		Industrial added value (s_12_)	New value-added to the production process of regional industrial enterprises	+	[27]
		Per capita disposable income (s_13_)	Changes in regional living levels	+	[17,27]
		Engel coefficient (s_14_)	Food expenditure as a share of total personal consumption expenditure	-	[18,28]
		Flood direct economic loss (s_15_)	Direct property damage from regional flooding disasters	+	[17,28]
		Degree of industrial structure optimization (s_16_)	Regional economic industry resource allocation efficiency	+	[46,47]
	Social subsystem (s_2_)	Population density (s_21_)	Population per unit land area	+	[10,26,27]
		Employment rate (s_22_)	Percentage of employed persons to the sum of employed persons and non-employed persons	+	[16,18]
		Urbanization rate (s_23_)	Percentage of urban population to the total population	+	[27,28]
		Drainage pipe length (s_24_)	The capacity of drainage pipes to discharge flood water	+	[17,26]
		Water construction investment (s_25_)	The amount of investment in the construction of water conservancy projects	+	[17,18]
		Policy inclination (s_26_)	Policy support for a region from a high-level government	+	[19,32]
	Natural environment Subsystem (s_3_)	Annual rainfall (s_31_)	The sum of the average monthly precipitation in a year	+	[10,25]
		Built-up area (s_32_)	The scale of a city in an area	+	[28,47]
		Green coverage (s_33_)	The ratio of the total area covered by greenery in the region to the total area of the region	-	[18,30]
		Sewage treatment rate (s_34_)	Regional capacity to treat wastewater	+	[10,27]
		Water quality compliance rate in water functional areas (s_35_)	The proportion of water functional areas that meet water quality standards to the water functional areas evaluated	+	[18]
		Water resources development and utilization degree (s_36_)	The ratio of exploited water resources to total water resources in a region	+	[18,32]

**Table 2:** Consistency Index (CI) table.

$r_{BW}$	**0.5**	**0.6**	**0.7**	**0.8**	**0.9**
CI (max ξ)	0	0.2	0.62	1.63	5.23

**Table 3:** $FBV^{\left(m\right)}$ and $FWV^{\left(m\right)}$ of subsystems and order parameters provided by the five experts.

	Experts	Subsystem	Order Parameter		
		(s_1_–s_3_)	(s_11_–s_16_)	(s_21_–s_26_)	(s_31_–s_36_)
$FBV^{\left(m\right)}$	*e*_1_	(0.5, 0.7, 0.6)	(0.6, 0.7, 0.6, 0.9, 0.5, 0.7)	(0.5, 0.7, 0.6, 0.7, 0.6, 0.9)	(0.5, 0.6, 0.6, 0.8, 0.7, 0.6)
	*e*_2_	(0.6, 0.5, 0.8)	(0.5, 0.6, 0.7, 0.8, 0.6, 0.7)	(0.6, 0.8, 0.5, 0.7, 0.7, 0.6)	(0.5, 0.6, 0.6, 0.7, 0.6, 0.6)
	*e*_3_	(0.5, 0.6, 0.7)	(0.6, 0.7, 0.6, 0.7, 0.5, 0.8)	(0.5, 0.6, 0.6, 0.7, 0.8, 0.6)	(0.5, 0.7, 0.6, 0.7, 0.6, 0.8)
	*e*_4_	(0.6, 0.5, 0.7)	(0.6, 0.8, 0.5, 0.7, 0.6, 0.6)	(0.5, 0.8, 0.7, 0.6, 0.6, 0.7)	(0.5, 0.7, 0.6, 0.8, 0.9, 0.6)
	*e*_5_	(0.7, 0.5, 0.8)	(0.6, 0.6, 0.7, 0.6, 0.5, 0.6)	(0.5, 0.7, 0.6, 0.8, 0.6, 0.6)	(0.5, 0.7, 0.6, 0.7, 0.8, 0.6)
$FWV^{\left(m\right)}$	*e*_1_	(0.7, 0.5, 0.6) ^T^	(0.8, 0.6, 0.7, 0.5, 0.9, 0.7) ^T^	(0.9, 0.7, 0.8, 0 6, 0.7, 0.5) ^T^	(0.8, 0.6, 0.6, 0.5, 0.6, 0.7) ^T^
	*e*_2_	(0.7, 0.8, 0.5) ^T^	(0.8, 0.7, 0.6, 0.5, 0.7, 0.6) ^T^	(0.7, 0.5, 0.9, 0.7, 0.6, 0.7) ^T^	(0.7, 0.7, 0.6, 0.5, 0.7, 0.7) ^T^
	*e*_3_	(0.7, 0.6, 0.5) ^T^	(0.7, 0.6, 0.7, 0.6, 0.8, 0.5) ^T^	(0.8, 0.7, 0.7, 0.6, 0.5, 0.7) ^T^	(0.8, 0.7, 0.6, 0.7, 0.7, 0.5) ^T^
	*e*_4_	(0.6, 0.7, 0.5) ^T^	(0.7, 0.5, 0.8, 0.6, 0.8, 0.7) ^T^	(0.8, 0.5, 0.7, 0.6, 0.6, 0.7) ^T^	(0.9, 0.7, 0.8, 0.6, 0.5, 0.7) ^T^
	*e*_5_	(0.7, 0.8, 0.5) ^T^	(0.7, 0.6, 0.6, 0.6, 0.7, 0.5) ^T^	(0.8, 0.6, 0.7, 0.5, 0.7, 0.7) ^T^	(0.8, 0.6, 0.6, 0.8, 0.5, 0.7) ^T^

Note: ^T^ represents the transpose of a matrix.

**Table 4:** Weights and CRs of subsystems.

Subsystem	*e*_1_	*e*_2_	*e*_3_	*e*_4_	*e*_5_	Average Weights
s_1_	0.478	0.337	0.478	0.299	0.278	0.374
s_2_	0.208	0.525	0.315	0.532	0.592	0.434
s_3_	0.315	0.138	0.208	0.168	0.130	0.192
$\xi^{*}$	0.003	0.009	0.003	0.040	0.019	
CRs	0.005	0.005	0.005	0.065	0.012	

**Table 5:** Weights and CRs of order parameters.

	*e*_1_		*e*_2_		*e*_3_		*e*_4_		*e*_5_		Average Weights
	Weights	CRs	Weights	CRs	Weights	CRs	Weights	CRs	Weights	CRs	
s_11_	0.216	0.010	0.297	0.005	0.191	0.005	0.168	0.017	0.193	0.081	0.213
s_12_	0.105		0.191		0.122		0.063		0.125		0.121
s_13_	0.170		0.122		0.191		0.284		0.155		0.184
s_14_	0.057		0.078		0.122		0.106		0.155		0.104
s_15_	0.318		0.191		0.297		0.212		0.288		0.261
s_16_	0.134		0.122		0.078		0.168		0.084		0.117
s_21_	0.299	0.015	0.183	0.015	0.270	0.031	0.288	0.031	0.253	0.018	0.259
s_22_	0.176		0.070		0.181		0.084		0.154		0.133
s_23_	0.140		0.304		0.145		0.125		0.154		0.174
s_24_	0.141		0.116		0.145		0.155		0.094		0.130
s_25_	0.178		0.145		0.078		0.193		0.154		0.150
s_26_	0.066		0.183		0.181		0.155		0.191		0.155
s_31_	0.278	0.025	0.236	0.048	0.304	0.015	0.327	0.031	0.302	0.017	0.290
s_32_	0.157		0.144		0.116		0.176		0.113		0.141
s_33_	0.157		0.178		0.183		0.109		0.226		0.170
s_34_	0.088		0.087		0.145		0.109		0.113		0.109
s_35_	0.126		0.178		0.183		0.058		0.067		0.122
s_36_	0.194		0.178		0.070		0.222		0.179		0.168

**Table 6:** Final ranking of FDR allocation indicators.

Subsystem	Subsystem Weights	Order Parameter	Local Weights of Order Parameter	Global Weights of Order Parameter	Ranking
Economic subsystem (s_1_)	0.374	s_11_	0.213	0.080	3
		s_12_	0.121	0.045	11
		s_13_	0.184	0.069	5
		s_14_	0.104	0.039	13
		s_15_	0.261	0.098	2
		s_16_	0.117	0.044	12
Social subsystem (s_2_)	0.434	s_21_	0.259	0.112	1
		s_22_	0.133	0.058	8
		s_23_	0.174	0.075	4
		s_24_	0.130	0.057	9
		s_25_	0.150	0.065	7
		s_26_	0.155	0.067	6
Natural environment subsystem (s_3_)	0.192	s_31_	0.290	0.056	10
		s_32_	0.141	0.027	16
		s_33_	0.170	0.033	14
		s_34_	0.109	0.021	18
		s_35_	0.122	0.024	17
		s_36_	0.168	0.032	15

**Table 7:** Change in weights of subsystems in the sensitivity analysis.

Subsystem	Weights of Other Subsystems as the Weight of the Social Subsystem (s_2_) Changes from 0.1 to 0.9								
s_1_	0.595	0.529	0.463	0.397	0.330	0.264	0.198	0.132	0.066
s_2_	0.1	0.2	0.3	0.4	0.5	0.6	0.7	0.8	0.9
s_3_	0.305	0.271	0.237	0.203	0.170	0.136	0.102	0.068	0.034
Total	1	1	1	1	1	1	1	1	1

**Table 8:** FDR allocation results of four cities under different allocation methods.

City	“PAG” Model			FBWM-TOPSIS	FBWM-Grey-TOPSIS
	Population	Area	GDP		
h_1_	0.1822	0.1786	0.1102	0.0748	0.1942
h_2_	0.2368	0.2034	0.1658	0.4341	0.3269
h_3_	0.3104	0.2152	0.2679	0.2074	0.2301
h_4_	0.2705	0.4027	0.4561	0.2836	0.2488

## Data Availability

The data that support the findings of this study are available from the corresponding author upon reasonable request.
